# Association of the Safe Routes to School program with school-age pedestrian and bicyclist injury risk in Texas

**DOI:** 10.1186/s40621-015-0038-3

**Published:** 2015-07-01

**Authors:** Charles DiMaggio, Joanne Brady, Guohua Li

**Affiliations:** 1 Department of Surgery, New York University School of Medicine, 550 First Avenue, New York, 10016 NY USA; 2 Department of Epidemiology, Mailman School of Public Health, Columbia University, 722 West 168 Street, New York, 10032 NY USA; 3 Department of Anesthesiology, College of Physicians and Surgeons, Columbia University, New York, NY USA; 4 Center for Injury Epidemiology and Prevention, Columbia University, New York, NY USA

## Abstract

**Background:**

Safe Routes to School (SRTS) is a federally funded transportation program for facilitating physically active commuting to and from school in children through improvements of the built environment, such as sidewalks, bicycle lanes, and safe crossings. Although it is evident that SRTS programs increase walking and bicycling in school-age children, their impact on pedestrian and bicyclist injury has not been adequately examined.

**Methods:**

We analyzed quarterly traffic crash data between January 2008 and June 2013 in Texas to assess the effect of the SRTS program implemented after 2009 on school-age pedestrian and bicyclist injuries.

**Results:**

The annualized rates of pedestrian and bicyclist injuries between pre- and post-SRTS periods declined 42.5% (95% confidence interval (CI) 39.6% to 45.4%) in children aged 5 to 19 years and 33.0% (95% CI 30.5% to 35.5%) in adults aged 30 to 64 years. Negative binomial modeling revealed that SRTS intervention was associated with a 14% reduction in the school-age pedestrian and bicyclist injury incidence rate ratio (IRR 0.86, 95% CI 0.75 to 0.98). The effect of the SRTS intervention on pedestrian and bicyclist fatalities was similar though smaller in magnitude and was not statistically significant (adjusted IRR 0.90, 95% CI 0.67 to 1.21).

**Conclusions:**

These results indicate that the implementation of the SRTS program in Texas may have contributed to declines in school-age pedestrian and bicyclist injuries.

## Background

Pedestrian injury is an important childhood public health issue. After many years of notable declines, the number of pedestrian fatalities across the US has increased nearly 5% between 2009 and 2011 (Patek and Thoma 2013). These numbers are mirrored by concomitant increases in cyclist fatalities (NHTSA 2011). This is in contrast to continued declines in both rates and frequencies of motor vehicle occupant fatalities and is reflected in the increasing proportion of traffic fatalities due to pedestrian injury. Because they tend to walk and bike more than other age groups, school-age pedestrians are particularly vulnerable for reasons of increased exposure and are subject to greater consequences when injury occurs due to anatomical factors like greater head to body ratio (Wazana et al. 1997). In 2011, pedestrians accounted for nearly 20% of traffic injury fatalities in children aged 5 to 9 years compared to 5% in adults (Kahn 2014). Pedestrian injury is the leading cause of traumatic brain injury for 5- to 9-year-olds (Hotz et al. 2009) and contributes to over half of all trauma-related hospital admissions for children in the US (Merrell et al. 2002). The consequences of pedestrian injuries in children extend beyond immediate trauma. An estimated 23% of children struck by cars will suffer psychological sequelae (Mayr et al. 2003). Concern about the potential dangers of walking and biking may contribute to childhood obesity and its attendant morbidities (Liu and Mendoza 2014; Pollack 2009).

In 2005, the US Congress funded the federal Safe Routes to School (SRTS) program as part of the federal Safe, Accountable, Flexible and Efficient Transportation Equity Act. The program was intended to encourage children to walk and bike to school and was allocated $612 million dollars for fiscal years 2005 to 2009 for state departments of transportation to build sidewalks, bicycle lanes, and safe crossings, improve signage, and make other improvements to the built environment to allow children to more safely travel to school (Safe Routes to School National Partnership 2009). As of 2012, departments of transportation in all 50 states and the District of Columbia had introduced safety improvements at 10,400 of the nations 98,706 elementary and secondary schools for a total cost of $1.12 billion, and nearly half of all available funds had been allocated for projects (Cradock et al. 2012). Legislation requires that the majority (70% to 90%) of funds be used for engineering and infrastructure projects like sidewalk construction, traffic calming measures, and capital improvements for pedestrian and bicycle access, with the remaining 10% to 30% used for education, encouragement, and enforcement activities (Levin Martin et al. 2009).

Eighty percent of projects are located in dense urban environments that are more likely to have higher proportion of disadvantaged and Latino students (McDonald et al. 2013; SRTS 2010). State departments of transportations have generally adhered to federal administrative guidance on the type and scope of interventions intended by the original legislation, with the large majority of proposed projects involving capital construction and engineering interventions (Cradock et al. 2012).

Additional studies have assessed the effect of SRTS programs in increasing active travel to school. Programs have had a demonstrable effect on travel behavior as measured by both self-report and socioecological models of public health interventions (Chriqui et al. 2012; Levin Martin et al. 2009). A nationally representative study of the impact of school-travel-related laws on active travel by school-age children concluded that in the relatively few states that have laws requiring traffic calming, there has been a demonstrable increase in active travel to school (Chriqui et al. 2012). Another study that looked at pre- and post-project active school-travel survey data at 53 schools in Mississippi, Wisconsin, Florida, and Washington found statistically significant increases in walking, from 9.8% of respondents in the pre-project period to 14.2% in the post-project period. While there were relatively smaller increases in bicycling (increased from 2.5% pre to 3.0% post), the researchers concluded that the projects were particularly effective at introducing bicycling to those communities where it was rare (Stewart et al. 2014).

Despite the importance of traffic injury to child health and the potential impact of SRTS programs on all pedestrian and bicyclist injury risks, fewer studies have assessed SRTS programs from the perspective of pedestrian and bicyclist injury control and prevention. Those that have addressed issues of pedestrian safety have assessed behaviors and perceptions linked to pedestrian safety, rather than crash and injury records (Boarnet et al. 2005) or consist of reviews of the existing literature rather than primary data analysis (Dumbaugh and Frank 2007). As part of a series of studies aimed at closing this research gap, we documented the safety benefit and cost-effectiveness of the SRTS program in New York City (DiMaggio and Li 2013; Muennig et al. 2014). Because our previous research evaluating the effectiveness of SRTS in reducing school-age pedestrian injury was limited to New York City, it may not be generalizeable to other geographic regions due to the urban environment, high population density, and special traffic patterns that may be unique to New York. In the present study, we evaluate the effectiveness of the SRTS program in reducing pedestrian and bicyclist injuries in school-age children in the state of Texas, which differs from New York City in traffic environments, population density, demographic characteristics, and other important aspects.

## Methods

Pedestrian crash data were obtained from the Texas Department of Transportation Crash Records Information System and consisted of individual-level police reports on pedestrian injuries in the state of Texas between January 2008 and June 2013. This information system includes all crash data collected from a Texas Peace Officer’s Crash Report (Texas Department of Transportation 2013), including roadway attributes and location data for crashes occurring on state highways. Variables included age and gender of pedestrians and bicyclists struck by motor vehicles, date, time, and factors contributing to the crash. The data includes investigations of all pedestrian and bicyclist injuries with some limited exceptions, e.g., incidents involving parked vehicles or trains. Information on Safe Routes to School projects in Texas was obtained from the National Center for Safe Routes to School (Safe Routes to School National Partnership 2014) and was used to determine the date when the funding was awarded to define pre- and post-intervention time periods. Decennial population counts at the state level were obtained from the US Census (United States Census Bureau 2010). Inter-census population estimates were obtained from the Texas State Library System (Texas State Library Archive 2013). There was an overall population increase of 2,154,823 or about 8.3% during the study period, which represents an approximate 1.3% increase for each year of the study. A log-log model was used to linearly extrapolate yearly population estimates (Gelman and Hill 2007).

Age was truncated at 109 years old as an upper cutoff. Two mutually exclusive age categories were created. School age was defined as 5 to 19 years old, inclusive. Adult age was defined as 30 to 64 years old, inclusive. For reasons of confidentiality, no month variable was included with the dataset. A year-quarter variable was created. We chose 2010 as the intervention year because the majority (244 of 313 or 78%) of intervention projects were funded in 2009. Analyses were based on all state-wide data, including districts that received funding after 2010 and those that did not.

Injury and fatality rates for the two age groups were annualized by dividing the quarterly injury counts by one quarter of the yearly population. Quarterly population-based injury and fatality rates for each age group were calculated and plotted, and we compared the average pre-intervention injury and fatality rates to the post-intervention rates for each age group. We calculated the difference between the average pre-intervention injury and fatality rates and the post-intervention rates for each age group with 95% confidence intervals for the change in the rate from one time period to the other (Aragon 2012).

A negative binomial model was fit to assess the effect of the post-SRTS intervention time period on the risk of pedestrian and bicyclist injury in children compared to adults. The rationale for this model was to discern the childhood injury and fatality trends independently of temporal trends as represented by the adult data. The final model was:

$$\begin{array}{*{20}l} {\text{log}}\!\left({\text{InjCount}}_{i}\right) \!&= \beta_{0} + \beta_{1}* \text{age group} + \beta_{2} * {\text{SRTS}}\\ &\quad\,+\, \beta_{3} *\! {\text{age group}} *\! {\text{SRTS}} \,+\, {\text{log(population)}} \end{array} $$

where,

InjCount_*i*_ is the count of pedestrian and bicyclist injury in quarter *i*, age group is a binary variable (1 for ages 5 to 19 years and 0 for ages 30 to 64 years), SRTS is an indicator of whether the injury occurred before or after the SRTS program was implemented (0 for January 2008 to December 2009, 1 for January 2010 to June 2013), and population is an offset variable that allows the exponentiated coefficients to be interpreted as incidence rate ratios (IRR).

The regression coefficients are interpreted as logarithms of the incidence density ratios for the risk of pedestrian and bicyclist injuries for different contrasts and combinations of age groups and time periods. With the other regression coefficients held to zero, then, the *β*_1_ coefficient is the estimated injury risk for school-age children compared to adults in the pre-SRTS time period. The *β*_2_ coefficient is the injury risk restricted to adults during the post-SRTS time period. The linear combination of *β*_1_+*β*_3_ is the injury risk in school-age children vs. adults during the post-SRTS time period. And, the combination of *β*_2_+*β*_3_ is the change in risk for school-age children after implementation of the SRTS program. In the model, we are most interested in the interaction term, *β*_3_, which is numerically the linear contrast of (*β*_2_+*β*_3_)−*β*_2_ and thus can be interpreted as the net effect of the SRTS program on the risk of pedestrian and bicyclist injury in school-age children when the temporal trend represented by adult injury data is removed.

The model was initially fit as Poisson and evaluated for model assumptions and goodness of fit. A chi-square test based on the residual deviance and degrees of freedom indicated overdispersion, and the final model was subsequently fit as a negative binomial process (Hinkelman 2012). A similar model was fit with annualized quarterly pedestrian and bicyclist fatalities as the outcome variable, and the coefficients interpreted similarly.

All data analyses were conducted in the R statistical programming language (R Development Core Team 2013). The study protocol was approved by the Columbia University Review Board as exempt.

## Results

**Figure 1:**
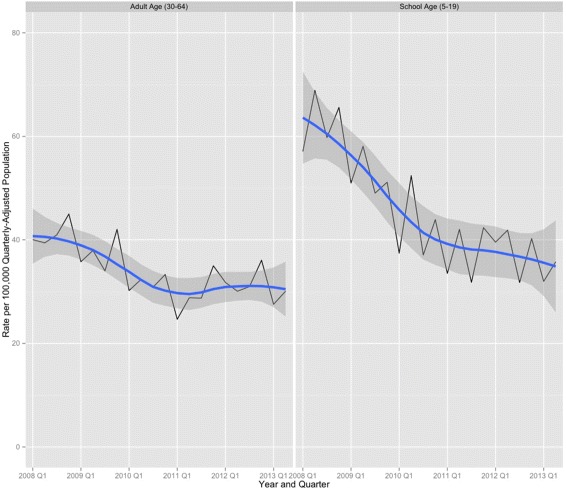
Adult vs. school-age injury rates over time. Time series plot with overlaid loess line and confidence envelope. Annualized rates of pedestrian and bicyclist injuries per 100,000 population, adults aged 30 to 64 years vs. children aged 5 to 19 years, Texas, January 2008 to June 2013.

A total of 52,042 pedestrians and bicyclists were struck and injured by motor vehicles in Texas between January 2008 and June 2013. The overall mean age for injured pedestrians and bicyclists was 32.8 years, with a median age of 29. For the population younger than 20, the mean and median values were 11.7 and 13 years old, respectively, with an interquartile range of 8 to 16. Bicyclists accounted for 14,677 (28.2%) of all injuries. For all ages, 33,535 (65.1%) of entries were coded male. In the school-age group, 9,565 (67.3%) were coded male. There were 2,714 deaths among all age groups (case fatality ratio = 5.2%) during the study period. Among the school-age group, there were 276 deaths, for a case fatality ratio of 1.9%. The yearly number of pedestrian and bicyclist fatalities in the study data was compared to and was the same as those reported for Texas in the US Fatality Analysis Reporting System (NHTSA 2014). Annualized pedestrian and bicyclist injury rates declined during the study period for both children and adults (Table 1 and Figure 1).

**Table 1:** **Pedestrian / bicyclists injury counts and rates per 100,000, adults (30-64 years) vs. children (5-19 years)**

	**Injury counts**		**Injury rates**	
**Quarter**	**Adult**	**School-age**	**School-age**	**Adult**
2008.1	912	658	57.07	40.03
2008.2	898	794	68.87	39.41
2008.3	933	689	59.76	40.95
2008.4	1,025	756	65.57	44.99
2009.1	906	653	50.98	35.79
2009.2	961	744	58.08	37.96
2009.3	861	628	49.02	34.01
2009.4	1,064	655	51.13	42.03
2010.1	850	533	37.45	30.22
2010.2	911	746	52.41	32.39
2010.3	869	528	37.10	30.89
2010.4	937	625	43.91	33.31
2011.1	763	525	33.53	24.66
2011.2	892	658	42.03	28.83
2011.3	890	498	31.81	28.76
2011.4	1,083	663	42.35	35.00
2012.1	1,082	681	39.54	31.79
2012.2	1,024	721	41.86	30.09
2012.3	1,055	547	31.76	31.00
2012.4	1,228	693	40.24	36.08
2013.1	1,032	606	31.99	27.56
2013.2	1,127	678	35.79	30.10

From the pre- to the post-SRTS intervention time period, there was a 42.5% decline in the annualized rates of school-age pedestrian and bicyclist injuries, from 57.3 injuries per 100,000 population during the pre-SRTS intervention time period (first quarter 2008 to fourth quarter 2009) to 32.9 injuries per 100,000 population in the post-SRTS intervention time period (first quarter 2010 to second quarter 2013). The 95% confidence interval (CI) for the change in the school-aged injury rate was 39.6% to 45.4%. In adults aged 30 to 64 years, there was a 33.0% decline in the annualized rates of pedestrian and bicyclist injuries, from 39.3 injuries per 100,000 population in the pre-intervention time period to 26.3 injuries per 100,000 population in the post-intervention time period (95% CI for percent decrease, 30.5% to 35.5%). The data for these results are not shown in the tables.

**Figure 2:**
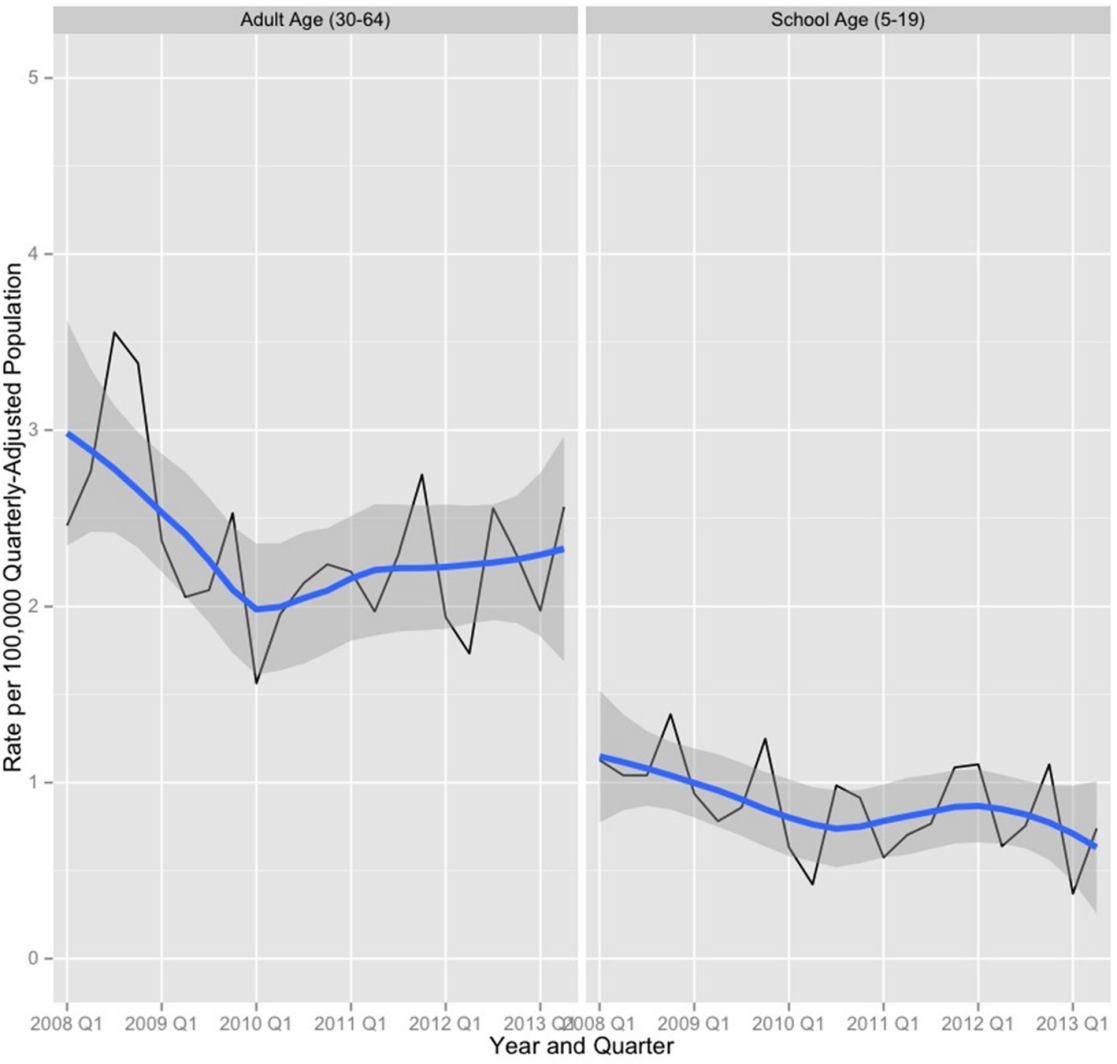
Time series plot with overlaid loess line and confidence envelope. Annualized death rates from pedestrian and bicyclist injuries per 100,000 population, adults aged 30 to 64 years vs. children aged 5 to 19 years, Texas, January 2008 to June 2013.

The annualized school-age pedestrian fatality rates decreased 37.1% (95% CI 14.9%, 59.4%) from 1.1 per 100,000 population before SRTS intervention to 0.7 per 100,000 population after SRTS intervention. The annualized adult pedestrian fatality rates decreased 29.6% (95% CI 19.7%, 39.5%), from 2.6 per 100,000 population before SRTS intervention to 1.6 per 100,000 population after SRTS intervention (Figure 2, data not shown).

In a negative binomial difference-in-differences model, implementation of the SRTS program in Texas was associated with a 14% reduction (adjusted IRR 0.86, 95% CI 0.75, 0.98) in pedestrian and bicyclist injury risk in school-age children. Specifically, the estimated IRR of pedestrian and bicyclist injury in school-age children vs. adults were 1.40 ($\phantom {\dot {i}\!}e^{\beta _{1}} = e^{0.34}$) and 1.21 ($\phantom {\dot {i}\!}e^{\beta _{1}+\beta _{3}=e^{0.34-0.15}}$) for the pre- and post-intervention periods, respectively (Table 2). In a separate negative binomial model of fatality data, implementation of the SRTS program in Texas was associated with a 10% reduction in the risk of pedestrian and bicyclist injury fatality in school-age children (adjusted IRR 0.90, 95% CI 0.67, 1.21, Table 3).

**Table 2:** Injury regression* injury results school-age (5-19 years vs. 30-64 years), intervention period (pre vs. post 2010), and interaction term

	**Estimated incidence rate ratio (95% CI)** ^**a**^
(Intercept)	0.00 (0.00, 0.00)
School-age	1.46 (1.30, 1.62)
Intervention period	0.78 (0.70, 0.85)
Intervention*age	0.86 (0.75, 0.98)

*Negative binomial statistical model of the effect of age group (school vs. adult), SRTS intervention time period (pre-post-2010) and interaction term on annualized quarterly pedestrian injury counts with population offsets. Texas pedestrian injuries January 2008 to June 2013.

^a^Exponentiated regression coefficient point estimate on relative risk scale with upper and lower 95% confidence limits.

**Table 3:** Fatality regression* results school-age (5-19 years vs. 30-64 years), intervention period (pre vs. post 2010), and interaction term

	**Estimated incidence rate ratio (95% CI)** ^**a**^
(Intercept)	0.00 (0.00, 0.00)
School-age	0.40 (0.31, 0.50)
Intervention period	0.82 (0.71, 0.94)
Intervention*age	0.90 (0.67, 1.21)

*Negative binomial statistical model of the effect of age group (school vs. adult), SRTS intervention time period (pre-post-2010) and interaction term on annualized quarterly pedestrian fatality counts with population offsets. Texas pedestrian injuries January 2008 to June 2013. ^a^Exponentiated regression coefficient point estimate on relative risk scale with upper and lower 95% confidence limits.

## Discussion

The national Safe Routes to School program has been hailed as a public health success story (Henderson et al. 2013), and SRTS interventions have been successful in addressing parent’s perceptions about their children’s safety getting to and from school (Cradock et al. 2012). These results indicate that those perceptions are supported by empiric evidence. Controlling for the temporal trend represented by the reduction in adult injuries, implementation of SRTS in Texas has led to a 14% reduction in pedestrian and bicyclist injury rates in school-age children, which translates into 404 fewer injuries to Texas school-age children each year. This finding is consistent with the experience of New York City, where implementation of SRTS resulted in a similar degree of reduction in pedestrian injuries in school-age children (DiMaggio and Li 2013). The safety benefit of SRTS programs reported in this study is likely to be conservative because the incidence rates based on population data do not take into account the increased exposure to walking and bicycling associated with SRTS programs and because SRTS programs were implemented in only about 443 of Texas’s estimated 9,932 schools (Safe Routes to School National Partnership 2009), while injury data from across Texas were included in our analysis. Nevertheless, these studies provide compelling evidence that children can be active and still be safe.

The built environment is directly tied to child pedestrian injury risk (DiMaggio and Li 2012). In a Toronto study that measured the association of the directly measured proportion of children walking to school with overall child pedestrian injury risk, a statistically significant crude incidence density ratio of 3.5 reduced to an incidence density ratio of 0.8 once built environment was taken into account (Rothman et al. 2014). Manipulating the built environment has been called a ‘logical but often overlooked’ area of injury control that may result in ‘the most successful interventions’ (Staunton et al. 2007). Recommended interventions include those that are commonly part of SRTS projects, such as separating play areas from roadways, improved visibility at intersections, conspicuous stop signs, enhanced pavement markings, and improved lighting (Schuurman et al. 2009).

The national Safe Routes to School program represents perhaps the largest expenditure on school-age pedestrian safety in the US history. It should come as no surprise that the program is associated with meaningful reductions in school-travel school-age pedestrian injuries. In this report, we add to our previous examinations of the program that demonstrated a nearly 40% decline in school-travel school-age pedestrian injuries in SRTS-targeted areas in a dense urban environment (DiMaggio and Li 2013), by looking at the effect of SRTS from a state-wide perspective in a setting that differs markedly from our previous analyses. While these analyses cannot demonstrate that the effect of SRTS safety improvements extends beyond the geographic areas to which they are targeted or that there was a more comprehensive benefit, the overall state average may actually obscure more dramatic improvements in the implementation areas than in the non-implementation areas. The injury control benefits of the SRTS program appeared to contribute to a decline in school-travel pedestrian injury across the state. This kind of effect can be expected to contribute to an even greater cost-effectiveness than the net societal benefit of $230 million and 2,055 quality-adjusted life years demonstrated in a more restrictive study of SRTS in New York City (Muennig et al. 2014).

These analyses, by including all SRTS projects in the state not just those involving capital construction, also include the benefits of educational programs. Those effects can be significant. In a survey of 699 children who participated in a bike safety educational program funded through SRTS, there were statistically significant post-intervention improvements in knowledge about bike safety, such as traffic rules and using helmets, with students scoring an average of 4 points higher on a 13-point scale on an instrument assessing knowledge about bike safety (Lachapelle et al. 2013).

Although the 14% reduction in pedestrian and bicyclist injuries that we report occurred during the *a priori* defined post-intervention time period, and most of the benefit appeared to accrue to the intended intervention population, school-age children, the results cannot be interpreted as causally due to SRTS. Similarly, while the higher net benefit for children included a 14% greater decline in injuries and a 10% greater decline in fatalities in children compared to adults, the demonstrated declines cannot be separated entirely from underlying secular trends. Indeed, declines in overall pedestrian injury rates predated the SRTS program, making it difficult to tease out or isolate the effects of this single program or intervention. Ascribing causation on pre-post comparisons can be subject to *post hoc ergo propter hoc* errors. Our use of difference-in-differences modeling was an attempt to address this issue through statistical means. Despite these efforts, it is difficult, if not impossible, to ascribe all the declines in school-travel school-age pedestrian and bicyclist injuries to the SRTS program.

The choice of adult data as the basis of comparisons is imperfect. There are certainly reasons beyond SRTS interventions why the pedestrian and bicycle injury experience of children differed from adults over time. Children may be more vulnerable to pedestrian and bicyclist injuries for reasons beyond simple exposure, including physical and cognitive development. There may also be contamination of effect, since the environmental changes would have an effect on both age groups. We chose the adult population for several reasons: to help control for trends, to distinguish the target population for SRTS interventions from a non-target population and to help differentiate between school and non-school-travel patterns. In this way, the adult comparison group helped establish the interpretation of the difference-in-differences analytic framework.

We chose a difference-in-differences analytic framework because the study period was relatively short, and we did not have adequate data to control for temporal trends using a more refined approach like interrupted time series. While difference-in-differences models have been proposed as a way to tease out policy impacts, in this case, data limitations limit interpretation to association rather than causality. Our choice of adults as a comparison was based on initial descriptive analyses looking at differing age groups. We believe the adult age group we chose differed sufficiently in terms of age and travel patterns from the target school-age group to be considered a separate population but could reasonably be expected to be sufficiently exposed to traffic as pedestrians.

Additional comparisons could have theoretically been based on sub-categories such as urban vs. rural or availability of mass transit, but the necessary data at smaller geographic units such as county or census tracts would not support that level of analysis. The choice of state-level analysis was driven in large measure by the quality and availability of the data. In previous analyses, we were able to geocode both the injury occurrence location and the site of SRTS interventions. We did not have sufficiently complete injury location data for these analyses. We note, that by including both intervention and non-intervention geographies at the macrolevel, we expect any demonstrated effects to be an underestimate.

Additional caveats are in order. Data limitations did not allow us, as in our previous studies, to spatiotemporally restrict our analyses to areas with SRTS interventions vs. areas without SRTS interventions just during school-travel times or to restrict our analysis to school-travel times. Also, in keeping with prior analyses, pedestrian and bicyclist injuries were analyzed together. This can be problematic and could underestimate the effect if interventions were primarily aimed at preventing pedestrian injuries. In fact, up to 30% of SRTS projects include educational activities to encourage children to bike to school (McDonald et al. 2013), and we believe our approach is consistent with the intent of SRTS to encourage both safe walking and bicycling to school. We also have found in that the coding of bicyclist vs. pedestrian injuries varies sufficiently across jurisdictions that combining the two modes of travel may introduce measurement error (DiMaggio and Li 2013) and believe the secondary analysis of administratively collected data to be too crude an instrument to validly separate pedestrian from bicyclist injuries. It would, though, be useful and informative to design future studies to examine the effect of SRTS interventions for each form of travel.

## Conclusions

Our data support the premise that the Safe Routes to School program, which is primarily a series of changes to the built environment, contributed to declines in school-age pedestrian injuries in Texas. We believe that expanding the kinds of interventions represented in SRTS to all schools can be expected to have important and sustained benefits to all pedestrians and that manipulating the physical environment is an effective, though often difficult and expensive, approach to pedestrian injury control.

## Abbreviations

CI: confidence interval
FARS: fatality analysis reporting system
IRR: incidence rate ratio
SRTS: Safe Routes to School
